# The association between oxidized low-density lipoprotein antibodies and hematological diseases

**DOI:** 10.1186/s12944-016-0360-y

**Published:** 2016-11-08

**Authors:** Hao Li, Da-qing Li, Xiang-xin Li, Lu-qun Wang

**Affiliations:** 1 Department of Hematology, Qilu Hospital, Shandong University, Jinan, 250012 China; 2 The Key Laboratory of Cardiovascular Remodelling and Function Research, Chinese Ministry of Education and Chinese Ministry of Health, Department of Cardiology, Qilu Hospital, Shandong University, 107# Wenhuaxi Road, Jinan, 250012 China

**Keywords:** Oxidized low-density lipoprotein, Autoantibody, Hematological diseases, oxLDL-IgG antibody, oxLDL-IgM antibody

## Abstract

**Background:**

The aim of the study is to compare the profiles of antibodies (IgM and IgG) against oxidized low-density lipoprotein (oxLDL) of hematological diseases.

**Methods:**

The serum antibodies of oxLDL-IgM and oxLDL-IgG for 446 cases with hematological diseases and 90 patients with primary hypertension and 90 healthy controls were measured by enzyme-linked immunosorbent assay (ELISA) in a cross-section survey. The association of serum oxLDL-LgM and oxLDL-IgG with hematological diseases was analyzed by multiple linear regression model.

**Results:**

Comparing with the hypertension or normal groups, the levels of TCH, TG, LDL-c, HDL-c, oxLDL, and oxLDL-IgG were lower and the levels of ADP and oxLDL-IgM were higher in the hematological diseases group. The levels of oxLDL-IgG antibodies titer were different among hematological diseases group. The results of correlation and multiple regression analysis showed that the seven hematological disease subgroups were positively related to the oxLDL-IgM antibody titer but negatively related to the oxLDL-IgG antibody titer, having been adjusted for potential confounding factors such as age, SBP, DBP, BMI, TCH, TG, ADP, oxLDL, HDL-c, LDL-C.

**Conclusions:**

Here we show that oxLDL-IgG antibodies titer were lower and of oxLDL-IgM titer were higher than hypertension and healthy individuals. Also oxLDL-IgG titer were different among hematological diseases group.

## Background

Oxidized low-density lipoprotein (oxLDL) consists of lipids, including cholesterol and oxysterols, phospholipids, oxidized fatty acids, aldehydes and lipid peroxides [1]. Studies have shown that oxLDL plays a major role in initiation and progression of atherosclerosis (AS) [2]. OxLDL has both proinflammatory and cytotoxic properties [3]. The relationship was firstly confirmed in the setting of hypercholesterolemia, with the highest IgM levels showing the lowest risk of coronary artery disease for the same level of hypercholesterolemia [4]. An epidemiological cohort of initially healthy subjects shows that IgG and IgM are not only independent predictors of coronary artery disease (CAD) events but also may modify CAD risk associated with elevated levels of oxidative biomarkers [5]. Interestingly, as early to be observed a high level of serum cholesterol may increase the risk of atherosclerosis, and a low level of serum cholesterol may increase the risk of cancer.

Recently, a genetic study showed the molecular basis of these connections, which indicated that lipid metabolism genes are important for transformation and are up-regulated in cancer and atherosclerosis. The genes of activation oxLDL and its receptor are a molecular connection between cancer and atherosclerosis [6]. Natural antibodies play an important role in the process of homeostatic inflammation, cell death and apoptotic cell clearance [7]. Natural IgM antibodies (nIgM) can remove oxLDL. The levels of nIgM specific for phosphoryl choline (anti-PC), were significantly higher in rheumatoid arthritis (RA) patients than osteoarthritis (OA) patients (*P* < 0.05), and that implicates the deficiency in immune responses to oxLDL which may contribute to the metabolic syndromes in the development of OA [8]. Positive immunoglobulin (Ig)G anticardiolipin antibody (IgGaCL) was the main component of oxLDL antibody. One research showed that the IgGaCL was associated with splanchnic vein thrombosis [9]. Moreover, some investigators found that the roles of anti-OxLDL antibodies (IgG/ IgM) are related to atherosclerosis and relevant diseases [10–13]. The human natural monoclonal IgM antibody SAM-6 is related to apoptosis of cancer cells [14]. We found that a decreased oxLDL and oxLDL-lgG and the elevated oxLDL-lgM serum levels may be related to the development and progression of esophageal squamous cell carcinoma [15].

As far as we know, a few papers reported the relationship between lipid and leukemia diseases. One report showed that the serum cholesterol, LDL, and HDL are significantly reduced in myelodysplastic syndrome (MDS) patients [16]. Another report showed that chronic lymphocytic leukemia (CLL) cells are antigen-experienced which may be derived from self-reactive natural Ab-producing B cells [17]. Our previous work found a decreased oxLDL-IgG and an elevated oxLDL-IgM serum levels in adult with acute myeloblastic leukemia (AML) [18]. A study with very enlightening significance showed that a 54-year-old female patient who had systemic lupus erythematosus with marrow fibrosis, when treated with high dose of steroids and intravenous immunogloblulin got remission with normalization of the bone-marrow [19]. So far little information of oxLDL antibodies is available about hematological diseases, especially for leukemia.

The presence of oxLDL and autoantibodies in several hematological diseases, particularly in those with very poor prognosis, and the occurrence of these diseases is still not clear. The main goal of the present study is to investigate the profiles of oxLDL, oxLDL-IgG and oxLDL-Ig antibodies in some hematological diseases and compared with healthy and primary hypertension patients.

## Methods

### Subjects

We carried out a cross-sectional study including 286 cases that were first diagnosed according to the WHO diagnosis criteria during the period from Jan. 2008 to Feb. 2013 in the department of hematology, Qilu Hospital, Shandong University. Among them, 98 cases were diagnosed with non-lymphatic leukemia, 60 cases with lymphatic leukemia, 52 cases with hemolytic anemia, 66 cases with immunothrombocytopenia, 57 cases with multiple myeloma, 58 cases with non-Kochkin’s lymphoma, 55 cases with myelodysplastic syndrome. Two groups were classified: the first group included 90 patients with primary hypertension diagnosed in the Division of Cardiology of the Qilu Hospital of Shandong University, and the second group comprised of 90 consecutive healthy normotensive persons serving as normal controls from a community screening examination of health care during the same period. Inclusion criteria were negative family history of CAD, myocardial infarction (MI), and stroke; nonsmoking status; and absence of hypercholesterolemia, hypertriglyceridemia, and diabetes mellitus. The Medical Ethics Committee of Qilu Hospital of Shandong University approved the study protocol, and written consent was obtained from each participant.

### Measurements

A survey of the characteristics of the subjects, involving name, gender, age, occupation, etc., was conducted using a questionnaire. Blood pressure was measured by mercury sphygmomanometer using Korotkoff phase V for diastolic, according to the WHO guidelines. Hypertension was defined as systolic pressure ≥140 mm Hg and/or diastolic pressure ≥90 mm Hg or use of any antihypertensive agents.

### Blood sampling and biochemical analysis

Venous blood samples were taken in the morning after an overnight fast for at least 12 h. The samples were taken from the femoral artery and immediately centrifuged at 3000 rpm for 10 min. The specimens were then frozen and kept at −80 °C until assay. Total cholesterol (TCH), high density lipoprotein cholesterol (HDL-c), triglycerides (TG) levels were measured with conventional methods. Low density lipoprotein cholesterol (LDL-c) was calculated by the Fried Ewald formula (LDL ‐ c) = TCH–(HDL ‐ c) ‐ TG/5. Serum adiponectin (ADP), oxLDL, oxLDL-IgG and oxLDL-IgM autoantibodies against-oxLDL measured by the enzyme-linked immunosorbent assay (ELISA) with commercially available kits (Adliteram diagnostic laboratories Ln., USA). The detail methods of oxLDL-IgG and oxLDL-IgM measurements were shown in our previous reports [15, 18]. OxLDL-lgG or oxLDL-lgM titers were calculated by constructing a standard curve according to the standards attached in the corresponding kits. These kits used the individually poached microplate strips that were coated with native or antigen; as a result, the antigen stability was shown to be longer than 4 months at least. In our work, the intra-assay and inter-assay reproducibility (coefficients of variation) of the assay were 3–5 % and 4–7 % respectively.

### Statistical analysis

Shapiro-Wilk’s W test of normality was used for data distribution analysis. The normality distributed data were expressed as the mean value ± SD. In the univariate analysis, multiple group comparisons were performed by one-way analysis of variance (ANOVA), and the significant differences between groups were assessed by Bonferroni test. A bivariate correlation analysis was conducted by the Spearman’s rank method. A generalized linear regression model was analyzed considering the main effects of the oxLDL-IgM and oxLDL-IgG titer and covariates (confounding factors) including gender, age, body index (BMI), systolic blood pressure (SBP), diastolic blood pressure (DBP), TCH, TG, HDL-c, LDL-c, and ADP. The subgroup of the hematologic diseases was denoted as below: 1 = cases with non-lymphatic leukemia, 2 = cases with lymphatic leukemia, 3 = cases with hemolytic anemia, 4 = cases with immunothrombocytopenia, 5 = cases with multiple myeloma, 6 = cases with non Kochkin’s lymphoma, 7 = cases with myelodysplastic syndrome. The Levene test for homogeneity of variance and Bartlett’s spherical test were carried out using the generalized linear model. In the analysis a two-tailed *P* value < 0.05 was considered as the statistical significance. All statistical analyses were done by SPSS19.0.

## Results

We first measured the oxLDL-IgG and oxLDL-IgG antibody titers in a small pre-experiment. On the basis of these results, we calculated the sample size about 30 ~ 50 cases in each group so as to detect the main difference of each antibody titers with a statistical power of 9 % at the 0.05 level (the calculation formula was *n* = 2((t_α/2_ + t_β_)^^ 2^σ^^ 2^)/δ^^ 2^, t_α/2_ = 1.96, β = 0.1, σ = 9.0, δ = 6 ‐ 8). The overall sample size of the study was at least 495 subjects. Finally we enrolled 626 subjects in this study.

Table 1 lists baseline characteristics of all subjects. The differences on the variables of age, systolic blood pressure and diastolic blood pressure are statistically significant among those groups. All these variables as covariates (confounding variables) are included in the multi linear regression model.

**Table 1:** Demographic and clinical features of the subjects in the study

Group	Gender (M/F)	Age	BMI	SBP*	DBP+
cases with non lymphatic leukemia	49/49	43.2 ± 18.18	23.2 ± 3.36	126.0 ± 16.33	68.9 ± 12.05
cases with lymphatic leukemia	25/35	31.1 ± 15.40	23.0 ± 3.60	123.1 ± 9.30	79.3 ± 6.2
cases with Hemolytic anemia	30/22	50.5 ± 20.40	23.7 ± 3.99	133.8 ± 19.62	66.1 ± 10.04
cases with immunothrombocytopenia	25/41	43.7 ± 17.37	22.5 ± 3.11	123.7 ± 13.54	76.9 ± 9.99
cases with multiple myeloma	32/25	57.3 ± 8.49	24.9 ± 2.73	135.0 ± 13.28	81.3 ± 12.24
cases with non Kochkin’s	22/36	48.4 ± 12.45	26.6 ± 4.36	109.7 ± 11.49	73.6 ± 8.97
cases with myelodysplastic syndrome	25/30	61.9 ± 13.57	25.1 ± 3.67	122.2 ± 13.10	67.7 ± 10.15
patients with primary hypertension	47/43	56.5 ± 10.87	25.8 ± 3.49	159.2 ± 17.15	94.2 ± 11.03
healthy normal persons+	40/50	45.4 ± 11.67	24.5 ± 3.35	115.3 ± 16.31	78.2 ± 10.03
F/*x* ^2^	10.724	16.662	4.766	40.045	30.959
P	0.218	<0.001	0.02	<0.001	<0.001

*: *SBP* systoilic blood pressure, +: *DBP* diastolic blood pressure

### Comparison of the levels of serum lipids between hypertension group and normal control group

As shown by Tables 2 and 3, the levels of serum TCH, TG, LDL-c, ADP and oxLDL-IgM in the hypertension group are higher than those in the normal control group. Comparing with the controls, the levels of HDL-c, oxLDL and oxLDL-IgG in the hypertension group are lower, in particular the difference of oxLDL level is statistically significant (*P* < 0.005).

**Table 2:** The distribution of lipid profile in the nine sub groups of the study

Serial number + group	CTH	TG	APD	HDL-C	LDL-C	oxLDL	oxLDL-IgG	oxLDL-IgM
① cases with non lymphatic leukemia	4.00 ± 1.018	1.64 ± 1.055	7.56 ± 9.976	0.68 ± 0.355	2.97 ± 0.991	31.48 ± 21.594	38.57 ± 19.483	18.70 ± 10.510
② cases with lymphatic leukemia	3.90 ± 1.358	1.44 ± 0.910	2.40 ± 1.089	1.14 ± 0.834	2.47 ± 1.158	21.76 ± 10.846	28.14 ± 19.185	20.24 ± 9.915
③ cases with Hemolytic anemia	3.07 ± 0.805	1.62 ± 1.352	6.58 ± 8.409	0.92 ± 0.481	2.13 ± 1.079	21.08 ± 9.871	31.98 ± 20.364	23.89 ± 9.081
④ cases with immunothrombocytopenia	4.50 ± 0.866	1.24 ± 0.419	4.59 ± 6.395	0.96 ± 0.956	3.28 ± 1.047	17.33 ± 4.716	45.96 ± 25.804	17.74 ± 9.512
⑤ cases with multiple myeloma	4.13 ± 1.829	1.60 ± 0.903	5.08 ± 7.687	0.44 ± 0.312	3.51 ± 1.526	25.39 ± 12.593	61.82 ± 33.245	17.97 ± 6.918
⑥ cases with non Kochkin’s	5.50 ± 1.273	2.16 ± 1.062	5.49 ± 8.018	1.24 ± 1.141	3.82 ± 1.073	24.47 ± 18.673	38.25 ± 19.241	21.58 ± 14.194
⑦ cases with myelodysplastic syndrome	3.83 ± 0.854	1.19 ± 0.352	14.25 ± 12.550	0.70 ± 0.799	2.89 ± 0.427	52.68 ± 33.884	21.93 ± 10.368	23.17 ± 12.745
⑧ patients with primary hypertension	5.78 ± 1.2223	1.58 ± 0.952	5.57 ± 7.457	1.04 ± 0.560	4.43 ± 1.401	25.83 ± 10.735	82.18 ± 10.117	10.31 ± 9.130
⑨ healthy normal persons	5.09 ± 0.568	1.35 ± 1.167	3.28 ± 4.068	1.52 ± 1.169	3.36 ± 1.281	38.91 ± 15.514	77.59 ± 7.165	8.82 ± 9.685
F	42.104	5.424	11.225	12.234	24.532	24.963	100.115	17.313
P	<0.001	<0.001	<0.001	<0.001	<0.001	<0.001	<0.001	<0.001

Abbreviation: *CTH* total cholesterol, *HDL-c* high density lipoprotein cholesterol, *TG* triglycerides, *LDL-c* low density lipoprotein cholesterol, *ADP* Adiponectin

**Table 3:** The post hoc Bonferroni’s test for individual group-wise comparisons in Table 2

(I) group	(J) group	CTH	TG	APD	HDL	LDL	oxLDL	oxLDL-IgG	oxLDL-IgM
①	②	1.000	1.000	0.002	0.015	0.305	0.020	0.032	1.000
	③	<0.001	1.000	1.000	1.000	0.001	0.015	1.000	0.144
	④	0.247	0.339	0.638	1.000	1.000	<0.001	0.545	1.000
	⑤	1.000	1.000	1.000	1.000	0.217	1.000	<0.001	1.000
	⑥	<0.001	0.046	1.000	0.001	<0.001	0.491	1.000	1.000
	⑦	1.000	0.192	<0.001	1.000	1.000	<0.001	<0.001	0.417
	⑧	<0.001	1.000	1.000	0.075	<0.001	0.858	<0.001	<0.001
	⑨	<0.001	1.000	0.020	<0.001	0.903	0.111	<0.001	<0.001
②	③	0.003	1.000	0.183	1.000	1.000	1.000	1.000	1.000
	④	0.137	1.000	1.000	1.000	0.004	1.000	<0.001	1.000
	⑤	1.000	1.000	1.000	<0.001	<0.001	1.000	<0.001	1.000
	⑥	<0.001	0.002	1.000	1.000	<0.001	1.000	0.058	1.000
	⑦	1.000	1.000	<0.001	0.101	1.000	<0.001	1.000	1.000
	⑧	<0.001	1.000	0.569	1.000	<0.001	1.000	<0.001	<0.001
	⑨	<0.001	1.000	1.000	0.186	<0.001	<0.001	<0.001	<0.001
③	④	<0.001	1.000	1.000	1.000	<0.001	1.000	0.003	0.057
	⑤	<0.001	1.000	1.000	0.056	<0.001	1.000	<0.001	0.118
	⑥	<0.001	0.146	1.000	1.000	<0.001	1.000	1.000	1.000
	⑦	0.017	0.672	<0.001	1.000	0.027	<0.001	0.237	1.000
	⑧	<0.001	1.000	1.000	1.000	<0.001	1.000	<0.001	<0.001
	⑨	<0.001	1.000	1.000	0.001	<0.001	<0.001	<0.001	<0.001
④	⑤	1.000	1.000	1.000	0.011	1.000	0.339	<0.001	1.000
	⑥	<0.001	<0.001	1.000	1.000	0.330	0.746	1.000	1.000
	⑦	0.048	1.000	<0.001	1.000	1.000	<0.001	<0.001	0.167
	⑧	<0.001	1.000	1.000	1.000	<0.001	0.082	<0.001	0.005
	⑨	0.034	1.000	1.000	0.001	1.000	<0.001	<0.001	<0.001
⑤	⑥	<0.001	0.074	1.000	<0.001	1.000	1.000	<0.001	1.000
	⑦	1.000	0.842	<0.001	1.000	0.179	<0.001	<0.001	0.315
	⑧	<0.001	1.000	1.000	<0.001	<0.001	1.000	<0.001	0.006
	⑨	<0.001	1.000	1.000	<0.001	1.000	<0.001	<0.001	<0.001
⑥	⑦	<0.001	<0.001	<0.001	0.011	0.001	<0.001	<0.001	1.000
	⑧	1.000	0.014	1.000	1.000	0.074	1.000	<0.001	<0.001
	⑨	1.000	<0.001	1.000	1.000	0.625	<0.001	<0.001	<0.001
⑦	⑧	<0.001	0.639	<0.001	0.443	<0.001	<0.001	<0.001	<0.001
	⑨	<0.001	1.000	<0.001	<0.001	0.694	<0.001	<0.001	<0.001
⑧	⑨	0.001	1.000	1.000	0.002	<0.001	<0.001	1.000	1.000

Note: ①,②,③,④,⑤,⑥,⑦,⑧,⑨ is the serial number of each subgroup indicator in Table 2

### Comparison of the levels of serum blood lipids between the hypertension group and the hematological diseases subgroups

Comparing with the hypertension group (⑧), the levels of serum TCH, TG, LDL-c, HDL-c, oxLDL, oxLDL-IgG and oxLDL-IgM are lower in the seven subgroups of patients with hematological diseases. For oxLDL-IgM except the immunothrombocytopenia subgroup (④), the levels of oxLDL-IgM titer are significantly higher than that in the hypertension group, while the levels of serum oxLDL-IgG in the seven subgroups (from ① to ⑦ shown in Table 2) of hematological diseases are lower than that in the hypertension group (*P* < 0.005).

### Comparison of the levels of serum blood lipids between the normal control group and the hematological diseases subgroups

In the Tables 2 and 3 are the results of multiple group comparison and post hoc Bonferroni’s test for individual group-wise comparison of the profiles of lipid in the study. Compared with ⑨, the levels of oxLDL-IgG antibodies titer significantly increased, and the oxLDL-IgM titer decreased, and except ⑥, plasma CTH levels significant decline in the subsets (from ① to ⑦) of the hematological diseases group (*P* < 0.001). Except ①, plasma oxLDL levels in ⑦ was higher and in the others subsets of the hematological diseases group was lower than in ⑨ (*P* < 0.05).

### Comparison of the levels of serum blood lipids in the seven subgroups of hematological diseases

In the seven hematological diseases group the levels of oxLDL-IgG antibodies titer were statistically significant in between-group comparisons including ①-②, ①-③, ②-⑤, ③-④, ③-⑤, ④-⑤, ④-⑦, ⑤-⑥ and ⑤-⑦ (*P* < 0.05); However, there was no significant difference for the levels of oxLDL-IgM antibodies titer (*P* > 0.05). Plasma oxLDL levels were statistically significant in between subsets comparisons including ①-②, ①-③, ②-⑦, ③-⑦, ③-⑦, ④-⑦ and ⑤-⑦ (*P* < 0.05).

A bivariate correlation analysis (Table 4) shows that the oxLDL-IgG antibody titer is positively related to age, BMI, systolic blood pressure (SBP), diastolic blood pressure (DBP), TCH, LDL-c and oxLDL but inversely related to adiponectin (ADP) and triglyceride (TG); Moreover, the levels of oxLDL-IgM antibody titer are inversely related to age, BMI, DBP, TCH and LDL-c and positively related to ADP and TG. It is noteworthy that there is an inversely relationship between the levels of oxLDL-IgM titer and the levels of oxLDL-IgM titer (*r* = −0.368, *P* < 0.01).

**Table 4:** The correlation coefficients of the measurement variables in the study

	Group	oxLDL-IgM	oxLDL-IgG	LDL-c	HDL-c	OxLDL	ADP	TG	TCH	SBP	DBP	BMI	Age
Group^a^	1.000												
oxLDL-IgM	−0.343^**^	1.000											
oxLDL-IgG	0.494^**^	−0.368^**^	1.000										
LDL-c	0.293^**^	−0.300^**^	0.301^**^	1.000									
HDL-c	0.169^**^	−0.084	−0.024	−0.225^**^	1.000								
OxLDL	0.187^**^	−0.008	0.237^**^	−0.058	0.090	1.000							
ADP	−0.182^**^	0.482^**^	−0.199^**^	−0.182^**^	0.002	0.062							
TG	−0.048	−0.031	−0.157^**^	0.302^**^	−0.221^**^	−0.085	−0.050	1.000					
TCH	0.417^**^	−0.379^**^	0.287^**^	0.802^**^	0.239^**^	0.046	−0.213^**^	0.299^**^	1.000				
SBP	0.051	−0.022	0.248^**^	0.106^*^	0.030	0.014	0.014	0.022	0.102^*^	1.000			
DBP	0.367^**^	−0.184^**^	0.390^**^	0.312^**^	0.106^*^	0.022	−0.184^**^	0.204^**^	0.432^**^	0.557^**^	1.000		
BMI	0.225^**^	−0.145^**^	0.227^**^	0.246^**^	−0.035	0.003	−0.025	0.219^**^	0.282^**^	0.205^**^	0.302^**^	1.000	
Age	0.258^**^	−0.137^**^	0.142^**^	0.156^**^	−0.111^*^	−0.099	−0.091	−0.003	0.122^*^	0.315^**^	0.108^*^	0.120^*^	1.000
Gender	−0.005	−0.056	0.003	0.124^*^	0.211^**^	−0.087	−0.054	−0.078	0.211^**^	−0.135^**^	0.086	−0.021	−0.099

^a^The variable group consists of nine sub groups that listed in the Table 4; *:*P* < 0.05, **:*P* < 0.01

Table 5 lists the correlation coefficients between the levels of serum blood lipid (including TCH, TG, LDL-c HDL-c, oxLDL and ADP) and the oxLDL-IgM /oxLDL-IgG antibodies titer. In the hypertension group, the levels of serum oxLDL-IgM titer are positively related to ADP and TG and inversely related to the levels of serum oxLDL; the levels of oxLDL-IgG antibody titer are positively related to the levels of serum LDL-c and TCH.

**Table 5:** The correlation coefficients between blood lipid terms and xLDL-IgG/oxLDL-IgM antibodies titer in each sub-groups

Group		IgG	LDL-c	HDL-c	OXLDL	ADP	TCH	TG
cases with non lymphatic leukemia,	IgM	−0.063	−0.259^**^	−0.099	0.267^**^	0.473^**^	−0.239^*^	−0.176
	IgG	1.000	0.133	−0.180	0.013	−0.082	0.003	−0.149
cases with lymphatic leukemia	IgM	−0.071	0.067	0.421^*^	−0.168	0.392^*^	0.244	−0.125
	IgG	1.000	0.110	−0.201	0.627^**^	−0.128	−0.306	0.107
cases with hemolytic anemia	IgM	−0.246	−0.611^**^	0.425^*^	−0.156	0.073	−0.832^**^	0.374
	IgG	1.000	0.226	0.140	0.911^**^	−0.112	0.436^*^	−0.358
cases with immunothrombocytopenia	IgM	0.067	−0.233	−0.167	0.517^**^	0.350^*^	−0.600^**^	−0.267
	IgG	1.000	0.233	−0.800^**^	−0.300	0.100	−0.383^*^	−0.400^*^
cases with multiple myeloma	IgM	−0.263	0.408^*^	0.490^*^	−0.207	0.503^**^	0.408^*^	0.531^**^
	IgG	1.000	−0.944^**^	−0.524^**^	0.296	−0.056	−0.944^**^	−0.821^**^
cases with non Kochkin’s lymphoma	IgM	−0.417^*^	−0.517^**^	0.683^**^	0.383^*^	0.351^*^	−0.183	−0.433^**^
	IgG	1.000	0.400^*^	−0.533^**^	0.450^**^	−0.243	−0.083	−0.067
cases with myelodysplastic syndrome	IgM	0.264	−0.178	−0.067	0.584^**^	0.962^**^	−0.042	−0.660^**^
	IgG	1.000	−0.624^**^	0.529^**^	0.257	0.222	0.363^*^	−0.186
patients with primary hypertension	IgM	0.213	−0.198	0.168	−0.273^*^	0.433^**^	−0.141	0.296^*^
	IgG	1.000	0.320^*^	−0.153	−0.065	0.030	0.276^*^	0.094
healthy normal control	IgM	−0.131	0.455^**^	−0.396^**^	0.193	0.449^**^	0.031	0.390^**^
	IgG	1.000	−0.198	0.330^*^	−0.010	−0.499^**^	0.015	−0.212

*: *P* < 0.05, **: *P* < 0.01

In the normal control group the levels of serum oxLDL-IgM antibody titer are positively related to LDL-c, ADP and TG and inversely related to the levels of serum HDL-c; however, the levels of serum oxLDL-IgG antibody titer are positively related to the levels of serum HDL-c and inversely related to the levels of serum ADP.

In the seven subgroups of hematological diseases the tendency of correlation is quite different. For example, in the non-lymphatic leukemia group, the level of the oxLDL-IgM antibody titer is positively related to the levels of serum TCH, oxLDL-c and ADP but inversely related to the levels of serum LDL-c; however, there is not significant correlation between oxLDL-IgG antibody titer among those study groups.

### Multiple linear regression model analysis

Table 6 lists that the outcome of two multiple linear regression model analyses, with the dependent variable of the oxLDL-IgM and oxLDL-IgG antibody titer respectively. Comparing with the normal controls, 7 subgroups of hematological diseases have the positive relationship to the levels of serum oxLDL-IgM antibody titers but the negative relationship to the levels of serum oxLDL-IgG antibody titers, having been adjusted for potential confounding variables such as age, SBP, DBP, BMI, TCH, TG, ADP, oxLDL, HDL-c, LDL-C and hypertension group.

**Table 6:** The main effects for the variables related to the dependent variable of oxLDL-IgM or oxLDL-IgG in the generated linear regression models^b^

Parameters in the regression model	Dependent Var oxLDL-IgM			Dependent Var oxLDL-IgG		
	B	Wald	P	B	Wald	P
Constant	13.281	7.294	0.007	69.359	52.451	<0.001
Gender (M/F)	0.054	0.003	0.956	5.797	8.663	0.003
cases with non lymphatic leukemia	5.418	7.315	0.007	−37.985	106.186	<0.001
cases with lymphatic leukemia	8.340	11.736	0.001	−47.171	110.637	<0.001
cases with Hemolytic anemia	11.541	18.090	<0.001	−44.355	71.523	<0.001
cases with immunothrombocytopenia	6.469	9.687	0.002	−26.003	39.866	<0.001
cases with multiple myeloma	8.833	15.391	<0.001	−16.732	13.011	<0.001
cases with non Kochkin’s	9.716	22.301	<0.001	−32.233	63.987	<0.001
cases with myelodysplastic syndrome	7.196	7.659	0.006	−57.709	161.408	<0.001
patients with primary hypertension	1.692	0.697	0.404	4.992	1.440	0.230
healthy normal persons^a^	0.0	.	.	0.0	.	.

^a^The baseline level is healthy normal persons group

^b^In the two regression models we adjusted for potential confounding factors including age, SBP, DBP, BMI, TCH, TG, ADP, oxLDL, HDL-c, LDL-C

## Discussion

In the present study, we found out that (1) the profiles of serum lipid including oxLDL-IgM and oxLDL-IgG are consistent with the reported results in the literature [4, 5], comparing with the normal controls; (2) the mean levels of the serum lipid including TCH, TG, LDL-c, HDL-c, oxLDL, oxLDL-IgG and oxLDL-IgM are lower and the levels of ADP and oxLDL-IgM are higher in the patients with seven types of hematological diseases, comparing with normal controls; (3) the change in the trend of the profiles of serum lipid is the same as that of the normal controls, comparing with the patients with hypertension. The results suggest that the association tendency between lipids and cardiovascular diseases is different from that between lipid and hematological diseases. At the same time the levels of oxLDL-IgG have a significant difference among the different hematological diseases, in particular for leukemia. This result is consistent with our previous report [18].

The immunoglobulin, the oxLDL-IgM and oxLDL-IgG antibodies, are the origin of autoantibody production of oxLDL. Therefore, it is reasonable to conclude that they probably play a more important physiological role in modulating the development of hematological diseases. In view of the molecular immunology, it’s well known that the molecule of the IgM antibody is relatively large and responds sensitively to the recent infectious agent; however, the molecule of the IgG antibody is relatively small and responds sensitively to the previous infectious agent, indicating the host with an ability of immunity to the agent.

To the best of our knowledge, no related work has been reported previously. In our previous studies we found that the cytotoxicity of oxLDL on vascular endothelial cells line was lower than that on esophageal cancer cell and leukemia cell lines, as well as in patients with squamous cell esophageal carcinoma with a higher level of the oxLDL-IgM antibody titer and lower level of the oxLDL-IgG antibody titer, comparing with the normal persons [15, 18, 20]. The concentrations of ox-LDL required to induce apoptosis are usually >20 μg/m; these concentrations are quite high and observed only in patients with acute coronary syndromes [21]. Low physiologic concentrations (<5 μg/ml) do not cause significant apoptosis, and are associated with the cell proliferation [22]. Our assay indicated that the cytotoxic effects of oxLDL on K562/AO2 and EC-9706 were significantly higher than that on cancer cells of HT29, OVCAR3 and OVCAR5, HeLa, MCF7, A549 and PC3, when all aforementioned cell lines treated by oxLDL with the doses ranging from 100 to 300 μg/ml inhibited cell proliferations at dose- and time- dependence [23]. Combined with the results of the present study, we put forward the following hypothesis: oxLDL inducing autoimmune response is not only involved in the development of atherosclerosis or other vascular disease, but also related in the process of cancer and other chronic diseases including hematological diseases.

Oxidation of lipoproteins and oxidative processes generally play an important role in the initiation and progression of atherosclerotic lesions and the main mechanism is associated with the inflammatory and immune responses [24]. Recently, Lopes-Virella MF & Virella G presented a review of the modified LDL including oxLDL auto antibodies in relation of the adaptive immune response in atherosclerosis (AS) [25]. We reviewed the literature reports and divided them into three categories: first hypothesis, oxLDL antibodies as an independent factors doctrine, some reports indicated that oxLDL and its autoantibody oxLDL-IgG can increase the risk of AS, but its autoantibody oxLDL-IgG may decrease the risk of AS [26]; Second hypothesis, some reports contradicted this conclusion [27], Third hypothesis, some reports suggested a humoral immune response to the modified LDL (mLDL) can be pathogenic rather than the protective factor [25, 28]. One relatively large sample clinic data showed that the titer levels of antibodies to oxLDL in the CAD patients without and with acute coronary syndromes had not significant differences [29]. So far, the mechanism of antibodies of oxLDL or other mLDL associated with AS is still unclear. The evidences supporting oxLDL antibodies as the independent predictor for AS are summarized as following:First, the IgG and IgM antibodies provide directly protective effect due to the combining immune complexes [30]. Second, the antibodies to oxLDL block the uptake of oxLDL by macrophages and prevent the foam cell formation [28]. Third, probably the oxLDLAbs directed against some oxidized-phospholipid epitopes but not against others play a major protective role [31]. Once activated oxLDL antigen, T cells secrete a variety of cytokines and Th1 and Th2 cells have been postulated to play different roles in promoting inflammatory response [32].

The IgG and IgM antibodies are produced mainly due to the modification forms of oxLDL, malondialdehyde-modified LDL (MDA-LDL) and advanced glycosylation end product-modified LDL (AGE-LDL) [32]. Freigang S. et al. found that the antiatherogenic effect of immunization of modified LDL is not primarily dependent on very high titers of antibodies but more likely to result from the activation of cellular immune responses [26]. Recently, the results of some studies also showed that oxLDL auto antibody can be the cellular immune responses in the pathogenic process of diseases [33–35]. OxLDL was lower, whereas oxLDL-Ab was higher in patients without cardiovascular disease (CVD) comparing with those with CVD [33]. The blood level of assessed antibodies was significantly higher in stroke patients than in control group. However, this phenomenon does not seem to protect patients against cerebrovascular events [34]. Recently a report found that a quiescent population of IgM+ plasma cells including oxLDL-specific IgM antibody secreting cells in bone marrow also sustained the elevated IgM antibody response in circulation [35]. One author reported that comparing with the patients (aged >65 years) with OxLDL-Ab level ≥200 arbitrary U/ml, the Ox LDL-Ab level <200 arbitrary U/ml Adjusted Hazard Ratio (HR) was 2.67 (95 % CI 1.58–4.52) for composite outcome (hospitalization and mortality). OxLDL-Ab level was the best indicator for both hospitalization and composite outcome [36]. It is excited that a new approach of determination OXLDL antibody component, a novel single-chain variable fragments (scFvs) γκ5, has been established and the γκ5 scFv can detect the modified LDL at very low concentrations [37]. This will promote the deep study of the relationship between oxLDL antibodies and diseases.

The main limitation of this study is due to small sample of patients with hematological diseases in cross-section design, and still needs a large sample in prospective study to get a strong evidence to support the idea. Also the mechanism of the oxLDL and its auto-antibodies involved the development of various hematological diseases needs to be elucidated in the future.

## Conclusions

Our data suggest that the trend of association between lipids and cardiovascular diseases is different from that between lipid and hematological diseases. Also that the common characters of the different hematological diseases with the oxLDL-IgG antibody titers were lower, while the oxLDL-IgM antibody titers were higher than the normal group. Finally there were statistically significant differences of the oxLDL-IgG antibody titers among those hematological diseases. The results of this study are useful for further study on etiology and serious complications of hematological diseases.

## Abbreviations

ADP: Adiponectin
AS: Atherosclerosis
CAD: Coronary artery disease
CTH: Total cholesterol
CVD: Cardiovascular disease
HDL-c: High density lipoprotein cholesterol
LDL-c: Low density lipoprotein cholesterol
MDL-LDL: Malondialdehyde-modified LDL
mLDL: Modified LDL
oxLDL-Ab: oxLDL antibody
TG: Triglycerides
